# Blue-stain fungus from the Jurassic provides new insights into early evolution and ecological interactions

**DOI:** 10.1093/nsr/nwaf160

**Published:** 2025-04-26

**Authors:** Ning Tian, Yongdong Wang, Fangyu Li, Zikun Jiang, Xiao Tan

**Affiliations:** College of Paleontology, Shenyang Normal University, China; Key Laboratory of Evolution of Past Life in Northeast Asia, Ministry of Natural Resources, China; Nanjing Institute of Geology and Paleontology, Chinese Academy of Sciences, China; College of Resources and Environmental Engineering, Guizhou University, China; Chinese Academy of Geological Sciences, China; College of Paleontology, Shenyang Normal University, China

## Abstract

Definitive fossil blue-stain fungi was documented from the Jurassic of China, extending the earliest fossil record of this fungal group by approximately 80 million years. The new finding reshapes the evolutionary timeline of blue-stain fungi, and provides new insights into the evolution of their ecological associations with wood-boring insects.

Blue-stain fungi constitute a distinctive group of wood-colonizing fungi which lack the ability to decompose wood lignocellulose, yet are capable of causing significant sapwood discoloration, particularly in coniferous trees [1]. Though these fungi are generally non-fatal to their hosts, they often accelerate tree mortality when associated with wood-boring insects [1]. Taxonomically, blue-stain fungi form a polyphyletic group consisting of several genera within the class Sordariomycetes of Ascomycota [2]. Molecular phylogenetic analyses suggest that Sordariomycetes diverged from other Ascomycota during the Late Paleozoic to early Mesozoic [3,4]. Though some fossils have been referred to Sordariomycetes [5,6], hardly anything is known about the geological occurrences of blue-stain fungi. It was not until 2022 that the first credible record of the fossil blue-stain fungi was reported from the Upper Cretaceous in South Africa [7]. Herein, we report a fossil blue-stain fungus colonizing on an insect-infested conifer wood *Xenoxylon phyllocladoides* Gothan from the Jurassic Tiaojishan Formation in western Liaoning Province, Northeast China. This finding extends the earliest fossil record of blue-stain fungi by approximately 80 million years, reshaping the timeline of evolution of this fungal group, and provides new insights into the evolution of their ecological associations with wood-boring insects.

The wood host is preserved as a silicified wood fragment anatomically characterized by xenoxylean radial pitting and window-like cross-field pits (Fig. S1). The presence of wood-boring holes indicates it was once infected by wood-consuming insects (Fig. S1a). Within the wood tissues, well-preserved fungal remains represented by filamentous hyphae were found in both the ray parenchyma cells and the tracheid lumen (Fig. 1). The hyphae, measuring 3.0–4.5 μm in diameter, are septate and exhibit a distinct dark coloration under light microscopy (Fig. 1a–c). These hyphae are frequently encountered within the ray parenchyma cells, where they can grow by passing through the cross-field pits or directly penetrating the longitudinal and cross walls of ray cells (Fig. 1d–f). The hyphae seem initially confined to the ray cells, but then extended into adjacent axial tracheids (Fig. 1e). The present fossil hyphae possess the capability to directly penetrate tracheid walls, facilitating their perpendicular growth through different tracheids. Such a process was clearly observed in both the tangential and radial sections of the wood host (Fig. 1e, g–j, l). Specifically, upon contacting the cell walls, the hyphae typically form an appressorium-like structure (Fig. 1e, g–j, l), which is often linked to a slimmer penetration peg. Subsequently, the hyphal peg traverses the walls of two adhering tracheids and enters the lumen of the adjacent tracheid (Fig. 1e and h); thereafter, the hyphae resume normal size in diameter (Fig. 1e and h). In most cases, the appressorium-like structures are visible under light microscopy, while the penetration pegs are concealed by the tracheid walls which the hyphae penetrate (Fig. 1g, i, j, l). The astonishing details of the pegs are occasionally discernible in some cases in the present fossil specimen from China (Fig. 1e and h). For those hyphae that perpendicularly traverse the tracheids, they often bifurcate at right angles to form series of lateral branches parallel to the tracheid (Fig. 1l). In the radial longitudinal sections of the wood host, hyphae colonizing the tracheid lumen are found to be capable of passing through the bordered pits (Fig. 1k), potentially facilitating their movement from one tracheid to another via these pit pairs. Of interest, some slender hyphae with a smaller diameter of 2.0–3.0 μm were also observed within the wood tissues, particularly in the tracheid lumina (Fig. 1m–o). A distinctive characteristic of these hyphae is the frequent occurrence of numerous globose chlamydospore-like structures measuring 4.0–7.0 μm in diameter (Fig. 1m–o). In contrast to the thicker hyphae, these slender hyphae seem to be hyaline rather than having dark pigment under light microscopy.

**Figure 1.**
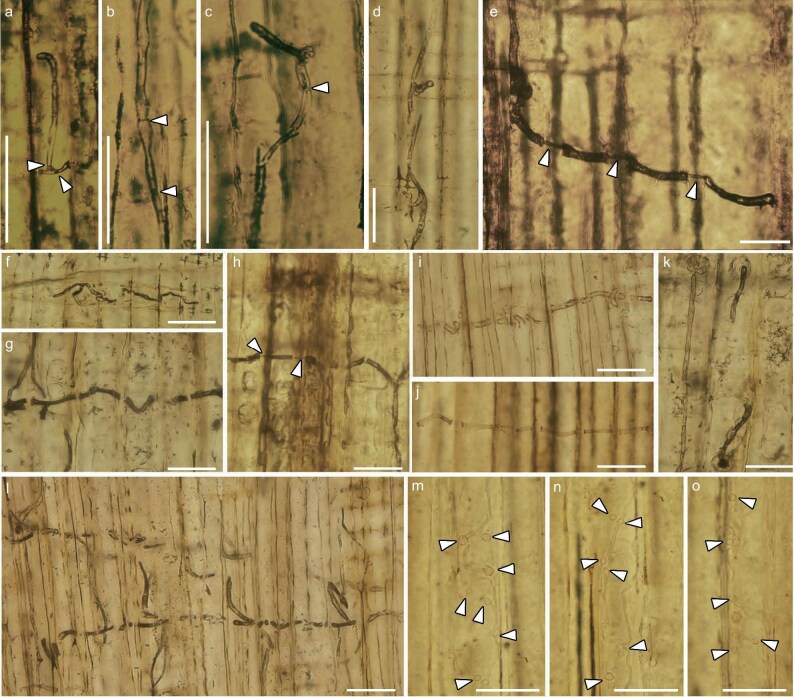
Blue-stain fungus in wood tissues of *Xenoxylon phyllocladoides* Gothan from the Jurassic of western Liaoning Province, NE China. (a–b, d–h, k) Radial section of the wood host. (c, i–j, l–o) Tangential section of the wood host. (a–c) Hyphae with septa (white arrow heads). (d) Hyphae growing in the cross-field zone. (e, h) Hyphae penetrating tracheid walls with appressorium-like structures and distinct hyphal pegs (white arrow heads). (f) Colonization of ray parenchyma cells by hyphae. (g, i–j, l) Hyphae horizontally penetrating the tracheid walls with appressorium-like structures. (k) Hyphae colonizing in the tracheid lumen, and passing through the bordered pits. (m–o) Slender hyphae within tracheid lumen with chlamydospore-like structures (white arrow heads). Bars: (a–d, f–n) 50 μm; (e) 25 μm.

The well-preserved septate hyphae with an absence of clamp connections enable us to place this new fossil fungus in the phylum Ascomycota. The present fungal fossil displays several traits akin to those observed in the modern blue-stain fungi. For the living blue-stain fungi, one of the most important diagnostic features of their hyphae is the formation of a distinct infection structure, consisting of the appressoria and penetration pegs, when penetrating the wood cells [7,8]. In contrast to wood-rotting fungi which enzymatically degrade lignocellulosic cell walls, blue-stain fungal hyphae employ mechanically driven penetration pegs to breach tracheid walls, due to the lack of enzymatic capacity to decompose lignocellulose [9]. Furthermore, pigmented thick-walled hyphae combined with preferential colonization patterns targeting ray parenchyma tissues are also crucial diagnostic criteria for identifying these fungi [7]. Microscopic examination reveals the present fossil hyphae as dark and thick-walled, indicative of pigmentation, a hallmark of contemporary blue-stain fungi which results in the discoloration of the wood host (Fig. S2) [8]. Additionally, the hyphae are quite abundant in the cross-field zone, implying a preferential colonization of ray parenchyma cells (Figs S2 and S3). Most importantly, the hyphae did not erode or decompose the tracheid walls, but directly penetrated them with appressoria and penetration pegs, which also align with the colonization pattern of extant ones in wood [9]. In addition, the occurrence of two morphological hypha types in the present fossil mycelium (i.e. the robust, darkly pigmented type and smaller hyaline type) was also found in modern blue-stain fungi from Europe [9] and an extant fungal sample examined herein from China (Figs S2 and S3). Based on this compelling evidence, it is conclusively determined that the Chinese fungal fossil exhibits an affinity with blue-stain fungi.

The extant blue-stain fungi form a polyphyletic group comprising multiple species within the genera *Ophiostoma* and *Ceratocystis*, which are currently classified under the orders Ophiostomatales and Microascales, respectively, within the class Sordariomycetes [2]. Due to the lack of enough diagnostic characters in the present fossil fungus, comprehensive comparisons between our fossil specimen and living genera of blue-stain fungi are not feasible. Molecular phylogenetic studies suggest that Sordariomycetes diverged from other Ascomycota lineages during the Late Paleozoic to early Mesozoic [3], potentially as early as the Middle Triassic [4]. A recent molecular clock analysis suggests that the crown age of existing Sordariomycetes dates to approximately 250 million years ago [10]. Within this class, the divergence of Microascales from other orders in Hypocreomycetidae is estimated to have occurred between 171 and 241 million years ago [11], while the divergence of Ophiostomatales from other Sordariomycetidae orders is inferred to have taken place between 130 and 188 million years ago [11]. From a fossil record perspective, the oldest reliable evidence of Sordariomycetes dates back to the Early Cretaceous [5,6], whereas, the earliest record of blue-stain fungi is from the Late Cretaceous, ca. 84 million years ago [7]. The present new finding from China with a Jurassic age represents the oldest documented occurrence of both blue-stain fungi and Sordariomycetes, indicating that the Sordariomycetes should have originated by at least the Middle to Late Jurassic (ca. 160 million years ago), thus extending their fossil record by approximately 80 million years.

The symbiotic relationship between blue-stain fungi and bark beetles is one of the most intensively investigated insect–fungal associations in forest ecosystems [12,13]. The dissemination of sexual spores of modern blue-stain fungi primarily relies on insect vectors, especially bark beetles and other wood-inhabiting insects [14]. It is widely acknowledged that the majority of blue-stain fungi within Ophiostomataceae are with specific bark beetle vectors in the weevil subfamily Scolytinae [15]; whereas, members of Ceratocystidaceae do not have specific insect vectors [1]. Both molecular biological and fossil evidence suggest that the calibrated origin time of Scolytinae dates back no earlier than the Early Cretaceous [16,17]. Given that the fungal remains from China are of Jurassic age, it is hypothesized that its spore dispersal vector was not Scolytinae but rather other wood-colonizing insects prevalent during that period, since, apart from bark beetles, some other wood-inhabiting beetles or arthropods may have also facilitated spore dispersal of blue-stain fungi [18]. Obviously, the symbiotic relationship between Scolytinae and modern blue-stain fungi, especially the Ophiostomatales, evolved after the Middle to Late Jurassic.

## Supplementary Material

nwaf160_Supplemental_File
