# Supplementary material for: Blue-stain fungus from the Jurassic provides new insights into early evolution and ecological interactions
Source: Natl Sci Rev. 2025 Apr 26;12(6):nwaf160. doi: 10.1093/nsr/nwaf160 (PMC12107238; doi:10.1093/nsr/nwaf160)
Supplement: nwaf160_Supplemental_File [file nwaf160_supplemental_file.doc]

**Supplementary Data:**

**The blue-stain fungi from Jurassic providing new insights into early evolution and ecological interactions**

Ning Tian^1,2*^, Yongdong Wang^3*^, Fangyu Li^4^, Zikun Jiang ^5^ and Xiao Tan^1^

^1^College of Paleontology, Shenyang Normal University, Shenyang 110034, China;

^2^Key Laboratory of Evolution of Past Life in Northeast Asia, Ministry of Natural Resources, Shenyang 110034, China;

^3^Nanjing Institute of Geology and Paleontology, Chinese Academy of Sciences, Nanjing 210008, China;

^4^College of Resources and Environmental Engineering, Guizhou University, Guiyang 550025, China;

^5^Chinese Academy of Geological Sciences, Beijing, 100037, China

*Corresponding authors. E-mail: [tianning84@163.com](mailto:tianning84@synu.edu.cn); [ydwang@nigpas.ac.cn;](mailto:ydwang@nigpas.ac.cn;)

**Supplementary data include the following files:**

Materials and methods

Wood host

Extant blue-stain fungi in modern Korean pine

Supplementary References

Supplementary Figure 1

Supplementary Figure 2

Supplementary Figure 3

**Materials and Methods**

The fossil wood specimen which bears fungal remains was collected from the Tiaojishan Formation in the Batuying Town of Beipiao City, western Liaoning Province, NE China. The Tiaojishan Formation in western Liaoning regions mainly comprises intermediate extrusive and pyroclastic rocks, intercalated with basic volcanic rocks and three plant-bearing sedimentary rock beds, i.e., the Dabangou Bed, the Shebudai Bed and the Taizishan Bed, ascendingly [1,2]. The present fossil wood was found in the Taizishan Bed. The age of the Tiaojishan Formation was traditionally considered to be late Middle Jurassic (e.g., ref [3,4]); however, some volcanic radiometric dating data proposed it with an age of 158 to 161 Ma, which indicates early Late Jurassic (e.g., ref. [5,6]). Herein, an age of late Middle Jurassic to early Late Jurassic is proposed for the Tiaojishan Formation, corresponding to the Callovian to Oxfordian.

The fossil material studied herein consists of a silicified wood fragment from which six thin sections were prepared, housed in the Palaeontological Museum of Liaoning (Shenyang, China) with registration number PMOL-B07242. The thin sections were prepared by standard methods, including cutting, grinding and polishing preparations [7]. Photographs were taken with the LAS V4.13 software adapted to a Leica DM4B Microscope and the Scope Image 9.0 (H3D) software adapted to a Yongxin BM2000 Microscope. For comparative study, a modern conifer wood sample (PMOL-B07243) of Korean pine (*Pinus koraiensis* Sieb. et Zucc.) with blue stained sapwood was collected from the artificial forest near the Dahuofang Reservoir in Fushun City of central Liaoning Province. The morphological details of the modern hyphae were observed under the Leica DM4B Microscope and a Hitachi S4800 Scanning Electron Microscope in the Paleontological Museum of Liaoning, Shenyang, China.

**Wood host**

The fossil wood host was preserved as a piece of silicified fragmented trunk with homoxylous secondary xylem. In the transverse section, distinct growth rings are present (Fig. S1a-c). Some holes which might be formed by wood-boring insects can be found (Fig. S1a). Tracheids of the early wood seem to be sheared (Fig. S1b-c). Bordered pits on the radial walls of tracheids are most circular, uniseriate, contentious or distant, and locally flattened (Fig. S1d-f). Cross-field pits are typical window-like, mostly one pit per field (Fig. S1g). The xenoxylean radial pitting and window-like cross-field pits allow it to be assigned to the common Mesozoic wood genus *Xenoxylon* Gothan. To date, over 20 species of *Xenoxylon* have been documented globally, spanning geological intervals from the Late Triassic to the Late Cretaceous [8]. Philippe et al. [9] established three morphogroups within this genus: the *X. latiporosum* group, the *X. phyllocladoides* group, and the *X. meisteri* group. Anatomical analysis of the present wood fossil permits its assignment to the *X. phyllocladoides* group, defined by contiguous or spaced circular-to-elliptical radial pits and localized xenoxylean pits [9]. This morphogroup currently comprises four recognized species: *X. hopeiense* Chang, *X. jakutiense* Shilkina, *X. phyllocladoides* Gothan, and *X. huttonianum* (Witham) Philippe et Haye [9]. The new specimen differs from *X. hopeiense*, by lacking axial parenchyma and biseriate radial pitting [10], and from *X. jakutiense* by the absence of Rims of Sanio [9]. Furthermore, cross-field pits in the earlywood zone of the present Chinese fossil wood display a width-to-height ratio of 1-2 (3):1, aligning diagnostically with *X. phyllocladoides* but contrasting with *X. huttonianum*, where this ratio reaches 4-5:1 [9]. Based on the diagnostic anatomical features described above, the present fossil wood can be confidently assigned to *X. phyllocladoides*, a common Mesozoic wood species widely recorded in the Jurassic and Cretaceous deposits of Europe, East Asia and Southeast Asia [9, 11].

**Extant blue-stain fungi in modern Korean pine**

Morphological details of fungal hyphae in modern blue**-**stained wood have historically been described in some publications [12-14]. Recently, a fresh light microscopic examination was given on a blue**-**stain fungus which colonized the wood of modern *Pinus strobus* [15]. In order to provide additional micrographs to compare with the present fossil fungi from China, a blue**-**stained sapwood sample of *Pinus koraiensis* (PMOL-B07243) was selected and examined under the Light Microscope and Scanning Electron Microscope. The selection of *P. koraiensis* mainly due to the fact that the species has window-like cross-field pitting which is similar to that of the present fossil wood sample.

The majority of fungal hyphae were observed in the cross-field zone of the pine wood, indicating an initial colonization of the ray parenchyma cells (Figs. S2a–l, S3). Hyphal branches originating from the ray cells were found to penetrate into neighboring tracheids (Fig. S2g–h) and subsequently extend along the tracheid lumina (Fig. S2m–n). Additionally, numerous hyphae were identified within both vertical and horizontal resin canals (Figs. S2o–p, S3c, f). The hyphae exhibited well-developed septa, which were clearly visible under light microscopy (Fig. S2). Similar to the fossil fungal remains described in this study, two distinct types of hyphae were identified within the fungal colonization zone (Figs. S2, S3). The thicker hyphae had a diameter of approximately 5.0 μm, while the slender hyphae measured 1.5–2.5 μm in diameter. These two types of hyphae were interconnected (Figs. S2, S3). Notably, chlamydospore-like structures were observed on hyphae colonizing the tracheids (Fig. S2q–s). No evidence of cell wall erosion was detected in the fungal-colonized regions of the wood host.

**Supplementary References**

1. Wang Y, Saiki K, ZhangW*et al.* *Prog Nat Sci* 2006; **26**: 222–30.
2. Zhang W, Yang XJ, Fu XP *et al.* *Rev Palaeobot Palyno* 2012; **183**: 50–60.
3. Zhang W, Zheng SL. Early Mesozoic fossil plants in western Liaoning, Northeast China. In: Yu X, Wang W, Liu X *et al.* (eds.). *Mesozoic Stratigraphy and Palaeontology of Western Liaoning (3)*. Beijing: Geological Publishing House, 1987, 239–68.
4. Zheng SL, Li Y, Zhang W *et al.* *Fossil woods of China*. Beijing: China Forestry Publishing House, 2008.
5. Chang SC, Zhang HC, Renne PR *et al. Earth Planet Sc Lett* 2009; **279**: 212–21.
6. Wang LL, Hu DY, Zhang LJ *et al*. *Chinese Sci Bull* 2013; **58**: 1346–53.
7. Hass H, Rowe NP. Thin section and wafering. In: Jones TP, Rowe NP (eds.). *Fossil Plants and Spores: Modern Technique*. London: Geological Society of London, 1999, 76–81.
8. Xie AW, Teng X, Wang YD *et al*. *Cretaceous Res* 2024; **154**: 105770.
9. Philippe M, Thévenard F, Nosova N *et al*. *Rev. Palaeobot Playnol* 2013; **193**: 128–40.
10. Chang CY. *Bull Geol Soc China* 1929; **7**: 243–55.
11. Li N, Zhang JP, Xing LD. *Hist Biol* 2020; **33**: 1686–1696.
12. Liese W. *Annu Rev Phytopathol* 1970; **8**: 231–58.
13. Ballard RG, Walsh MA, Cole WE. *Can J Bot* 1984; **62**: 1724–29.
14. Eriksson KE, Blanchette RA, Ander P. 1990. Biodegradation of Hemicelluloses. In: Eriksson KL, Blanchette RA, Ander P. *Microbial and Enzymatic Degradation of Wood and Wood Components. Springer Series in Wood Science*. Berlin, Heidelberg: Springer, 1990, 225–333.
15. Strullu-Derrien C, Philippe M, Kenrick P *et al. New Phytol* 2022; **233**: 1032–37.

**
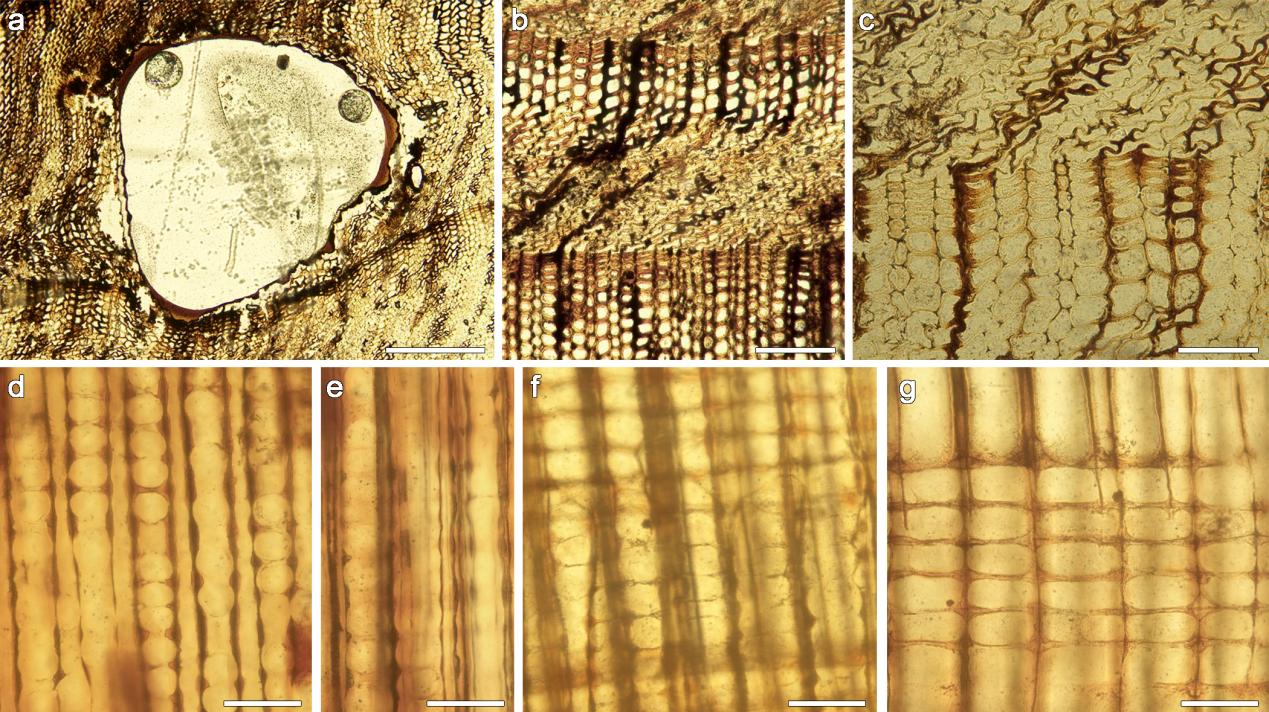
Figure S1 Anatomical details of the fungus-bearing *Xenoxylon phyllocladoides* Gothan from the Jurassic of western Liaoning Province, NE China.**

(a) Transverse section, a probable insect-boring hole. (b) Transverse section, distinct growth rings with collapsed early wood. (c) Transverse section, details of the growth ring, the sheared early wood tracheids and thick-walled late wood tracheids. (d) Radial section, uniseriate distant bordered pits. (e-f) Radial section, uniseriate continuous flattened bordered pits. (g) Radial section, window-like cross-field pits. Bars: (a) 500 μm; (b) 200 μm; (c) 100 μm; (d-g) 50 μm.


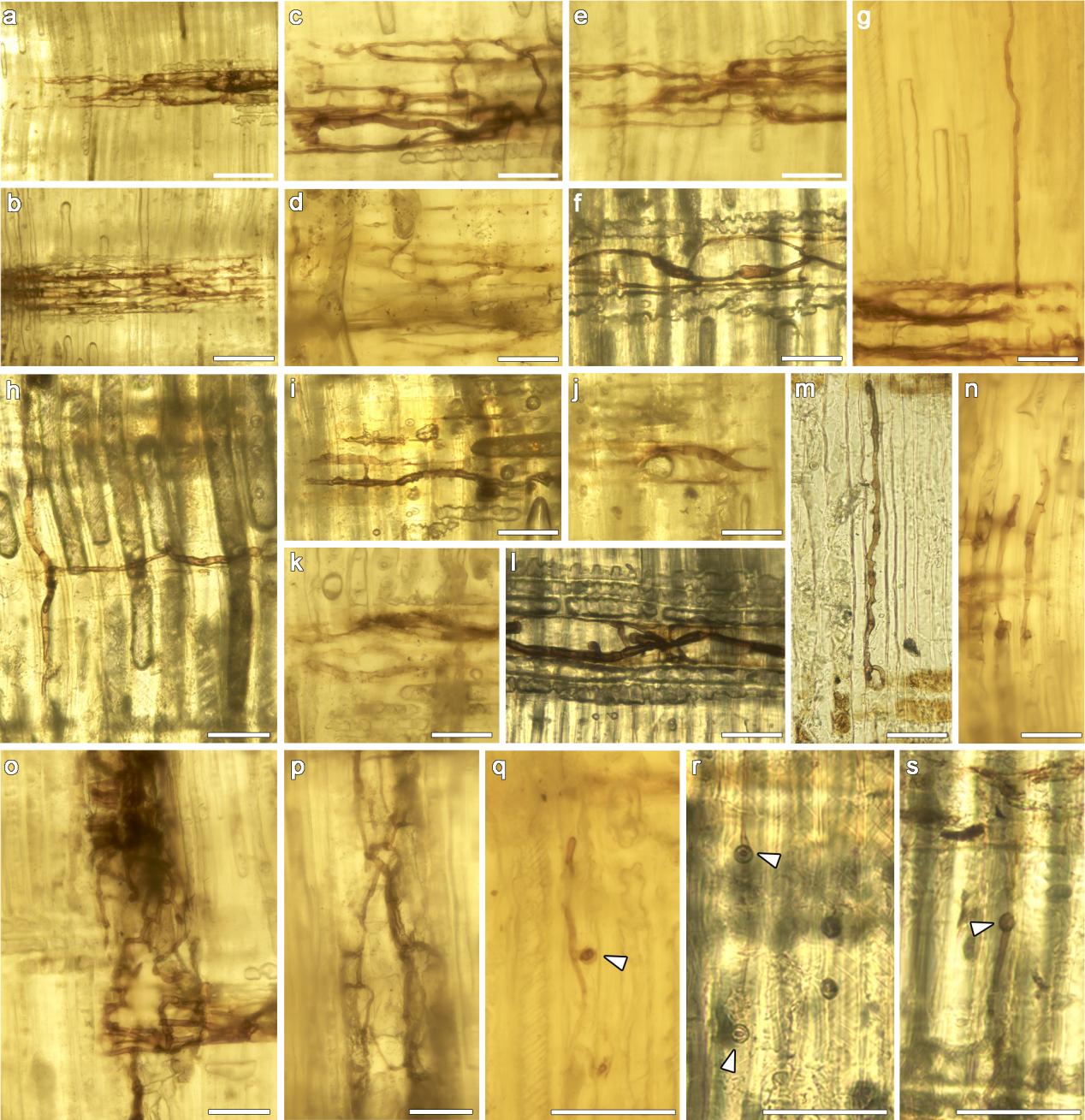


**Figure S2 Modern Blue-stain fungus in wood tissues of *Pinus koraiensis* Sieb. et Zucc.**

(a-s) Radial section of wood. (a-b) Colonization of ray parenchyma cells by hyphae. (c-e, i-l) Detail of septa hyphae in ray parenchyma. (f) Hyphae with varying diameter in ray cells. (g-h) Hyphae intruding into tracheids from ray cells. (m-n) Details of septa hyphae in tracheid lumen. (o-p) Septa hyphae within resin canal. (q-s) Chlamydospore-like structures (white arrow heads) born on the hyphae within the tracheid lumen. Bars: (a-b) 100 μm; (c-s) 50 μm.


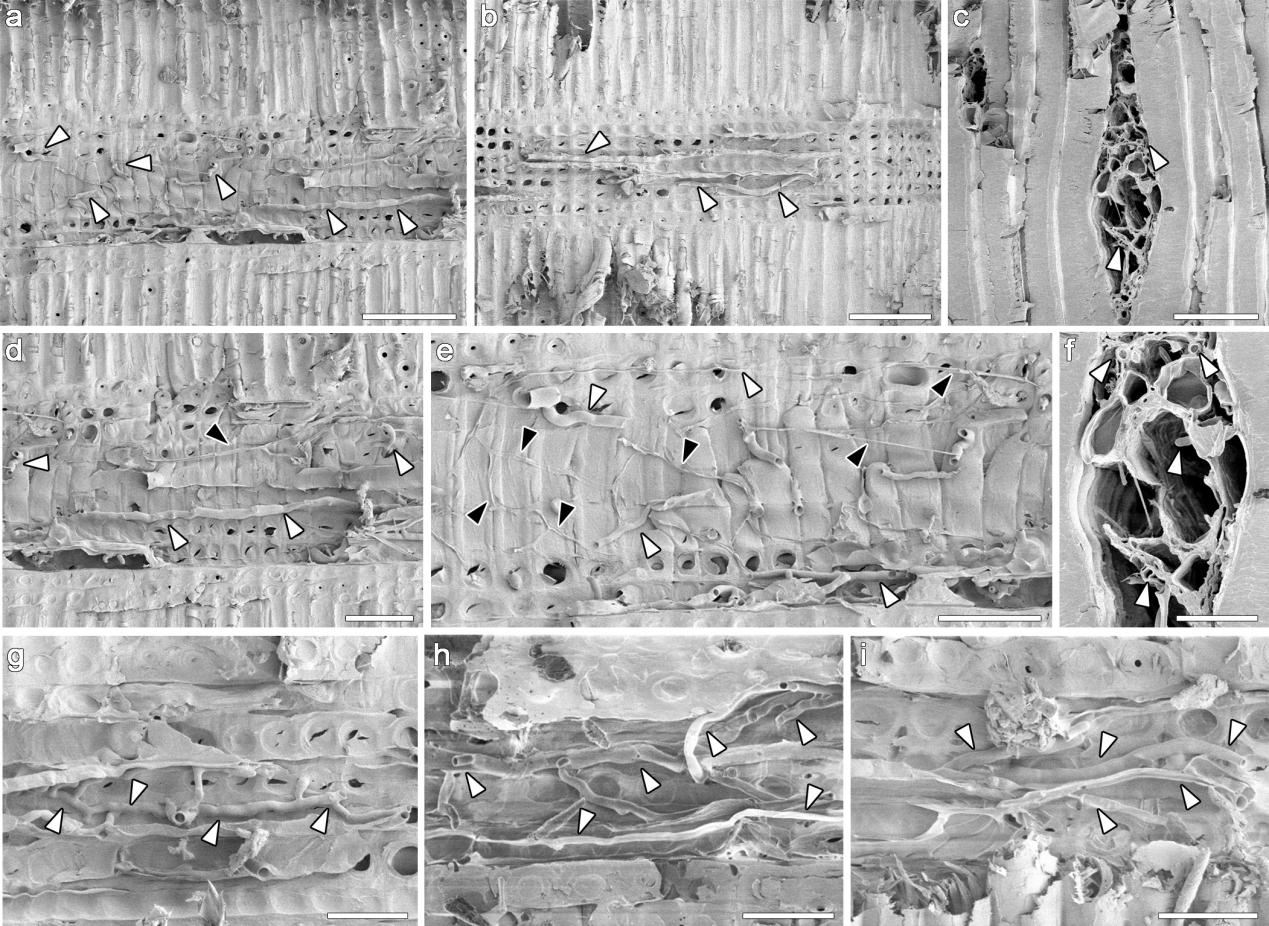


**Figure S3 SEM observation of modern blue-stain fungus in wood tissues of *Pinus koraiensis* Sieb. et Zucc.**

(a-b, d-e, g-i) Radial section of wood; (c, f) Tangential section of wood. (a-b) Colonization of ray parenchyma cells by hyphae (white arrow heads). (c) Hyphae colonizing the ray cells and horizontal resin duct (white arrow heads). (d-e) Robust hyphae (white arrow heads) and slender hyphae (black arrow heads) growing through ray cells or penetrating the cross-field pits. (f) Enlargement of hyphae (white arrow heads) in the ordinary ray cells parenchyma cells and the larger epithelium cells of the resin canal. (g-i) Details of hyphae (white arrow heads) in the ray cells. Bars: (a-b) 100 μm; (c, e) 50 μm; (d) 20 μm; (f, g) 30 μm.
